# The Biology of Lysosomes: From Order to Disorder

**DOI:** 10.3390/biomedicines11010213

**Published:** 2023-01-14

**Authors:** Olga Amaral, Mariana Martins, Ana Rita Oliveira, Ana Joana Duarte, Inês Mondragão-Rodrigues, M. Fátima Macedo

**Affiliations:** 1 Departamento de Genética Humana, Unidade de Investigação e Desenvolvimento, Instituto Nacional de Saúde Ricardo Jorge (INSA), 4000-055 Porto, Portugal; 2 Centro de Estudos de Ciência Animal (CECA, ICETA), Universidade do Porto, 4485-661 Porto, Portugal; 3 Laboratório Associado para Ciência Animal e Veterinária (AL4AnimalS), 1300-477 Lisboa, Portugal; 4 Departamento de Ciências Médicas, Universidade de Aveiro, Campus Universitário de Santiago, Agra do Crasto, Edifício 30, 3810-193 Aveiro, Portugal; 5 Instituto de Ciências Biomédicas Abel Salazar (ICBAS), Universidade do Porto, 4050-313 Porto, Portugal; 6 CAGE, Instituto de Investigação e Inovação em Saúde (i3S), Universidade do Porto, Rua Alfredo Allen, 208, 4200-135 Porto, Portugal

**Keywords:** lysosome, endocytic pathway, lysosome biogenesis and function, lysosomal disease

## Abstract

Since its discovery in 1955, the understanding of the lysosome has continuously increased. Once considered a mere waste removal system, the lysosome is now recognised as a highly crucial cellular component for signalling and energy metabolism. This notable evolution raises the need for a summarized review of the lysosome’s biology. As such, throughout this article, we will be compiling the current knowledge regarding the lysosome’s biogenesis and functions. The comprehension of this organelle’s inner mechanisms is crucial to perceive how its impairment can give rise to lysosomal disease (LD). In this review, we highlight some examples of LD fine-tuned mechanisms that are already established, as well as others, which are still under investigation. Even though the understanding of the lysosome and its pathologies has expanded through the years, some of its intrinsic molecular aspects remain unknown. In order to illustrate the complexity of the lysosomal diseases we provide a few examples that have challenged the established single gene—single genetic disorder model. As such, we believe there is a strong need for further investigation of the exact abnormalities in the pathological pathways in lysosomal disease.

## 1. Introduction

From waste removal system to key cellular signalling and energy metabolism component, it is clear that the understanding of the lysosome has thoroughly evolved since its discovery by de Duve in 1955 [1].

It is well established that this membrane-associated cytoplasmic organelle has a fundamental role in the digestion of macromolecules. As such, the lysosomes participate in the breakdown of extracellular and intracellular components delivered to them through endocytosis or autophagy, respectively [2]. In addition, this organelle participates in the regulation of energy metabolism, as it possesses signalling molecules to sense nutrient availability, such as mammalian target of rapamycin complex 1 (mTOR) and transcription factor EB (TFEB), that can increase lysosomal gene expression [2].

Lysosomal functions can be altered through diverse gene defects, which result in severe negative repercussions giving rise to lysosomal diseases (LDs). The term “lysosomal disease” is commonly used to refer to lysosomal storage diseases, where there is accumulation of undigested or partially digested macromolecules [3]. For a more correct understanding of the disease’s pathological mechanisms, we will describe the biology of the lysosome and explain how its dysfunction can lead to disease.

Traditionally, LDs are classified according to the substance that is abnormally accumulated in the lysosome—as mucopolysaccharidoses, mucolipidoses, sphingolipidoses, oligosaccharidoses, and neuronal ceroid lipofuscinoses [4,5]. However, in most LDs there can be more than one accumulated compound [3] or a considerable secondary accumulation. As such, this calls into question the current classification system [6,7,8] and led us to classify and characterize LDs using the protein deficiencies that cause them. We highlighted a lysosomal hydrolase deficiency, focusing on Gaucher’s disease (GD, MIM 230800; 230900; 231000); an integral membrane protein deficiency, for which Danon Disease (DD, MIM 300257) is an example; a lipid and ion transporters deficiency, such as mucolipidosis IV (MIM 252650); two enzyme modifiers, including an activator deficiency, GM2 Gangliosidosis (AB variant, MIM 272750) [3] and a posttranslational modifier, multiple sulphatase deficiency (MSD, MIM 272200). We believe this article will broaden horizons in the comprehension of the biology and pathology of the lysosome.

## 2. The Ordered Lysosome

### 2.1. Lysosome Biogenesis

The lysosome is a dynamic organelle, whose biogenesis requires the combined efforts of the endocytic and biosynthetic pathways. In the biosynthetic pathway, lysosomes are formed and acquire their necessary components, such as newly synthesized proteins. The lysosomes then fuse with membrane vesicles from one of three routes: endocytosis, autophagy, or phagocytosis. Through endocytosis, extracellular macromolecules are taken up into the cell to form membrane-bound vesicles—endosomes—that will fuse with lysosomes. In autophagy, old organelles and non-functioning cellular material are enveloped by internal membranes, which fuse with lysosomes. Lastly, in phagocytosis, specialized cells (e.g., macrophages) engulf large extracellular particles and target them for lysosomal degradation. The majority of the products of lysosomal digestion (e.g., amino acids and nucleotides) are recycled and used for the synthesis of new cell components [9].

#### 2.1.1. The Biosynthetic Pathway

Lysosome biogenesis entails a continuous restocking of its components, such as the soluble hydrolases and membrane proteins. These must be transported along the biosynthetic pathway, which encompasses the endoplasmic reticulum (ER), the Golgi complex, the *trans*-Golgi network (TGN), the plasma membrane, and the endosomes. The transport of lysosomal proteins is made through vesicles and requires a set of sorting signals and recognition proteins [9].

**Figure 1:**
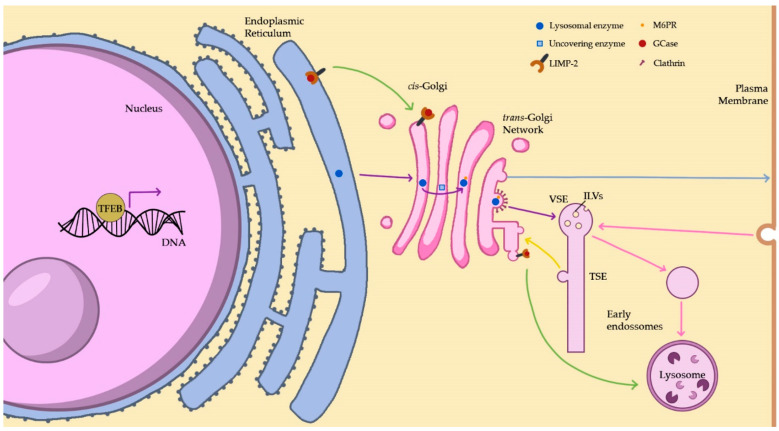
The lysosomal biosynthetic and endocytic pathways. Under starvation, TFEB is translocated to the nucleus and binds genes coding for lysosomal proteins, upregulating protein synthesis and initiating the biosynthetic pathway (purple arrows). Proteins are transported to the ER lumen. The proteins then travel to the *cis*-Golgi, some proteins require a membrane protein for this transport, like the β-Glucocerebrosidase (GCase) LIMP-2 mediated-transport (green arrows). In the *cis*-Golgi, the uncovering enzyme uncovers the mannose 6-phosphate (M6P) sugar in lysosomal proteins. Lysosomal proteins then arrive to the TGN, where Mannose 6-phosphate receptors (M6PR) recognize the M6P tag and are packed into clathrin-coated vesicles. Lysosomal proteins reach early endosomes (EE) and dissociate from M6PR and remain in the endosomal lumen. The M6PR can be recycled, returning to the TGN by the endosome to TGN carriers (yellow arrows). Endosomal intermediates transport the lysosomal proteins to the lysosomes through the endocytic pathway (pink arrows). Other proteins that don’t have specific target characteristics, may follow the constitutive secretory pathway to the plasma membrane (blue arrows) and reach lysosomes by endocytosis. VSE—vacuolar sorting endosome; ILVs—intraluminal vesicles; TSE—tubular sorting endosome.

The lysosomal proteins are synthesized through the coordinated transcription of the genes that encode them, a process that is crucial for lysosome biogenesis [10]. Many genes coding for lysosomal enzymes and lysosomal membrane proteins possess a coordinated lysosomal expression and regulation (CLEAR) element [11]. This element is a palindromic ten base pair GTCACGTGAC motif in the gene promoter region that can bind the transcription factor EB (TFEB). In its nonactive state, TFEB is highly phosphorylated, but under starvation or lysosomal dysfunction conditions, TFEB becomes dephosphorylated and is quickly translocated to the nucleus. This results in upregulation of lysosomal protein synthesis, such as acid hydrolases and other proteins found within lysosomes as well as lysosomal membrane proteins, thus, increasing lysosomal function [12] (Figure 1). TFE3 (transcription factor E3), another member of the MiTF/TFE family, acts in a similar manner to TFEB. In cells subjected to nutrient starvation, TFE3 is transported to the nucleus after inactivation of mammalian target of rapamycin complex 1 (mTORC1). It has also been found that TFE3 promotes the expression of genes associated with autophagy and lysosomes, also stimulating lysosomal biogenesis. These results indicate that cells rely on TFEB and TFE3 to control lysosomal homeostasis, with TFE3 serving as a major regulator of this process [13].

The biosynthesis of soluble lysosomal hydrolases starts with the insertion into the lumen of the rough endoplasmic reticulum (RER) due to the presence of a signal sequence in the N-terminal region. Inside the RER, the signal sequence is cleaved and core glycosylation occurs on selected asparagine residues on the nascent protein [14].

Furthermore, the soluble lysosomal hydrolases travel by vesicular transport to the *cis*-Golgi complex (Figure 1), where one of two things can happen: their oligosaccharide chains can be trimmed and modified by adding complex sugar residues (ex. galactose, N-acetyl neuraminic acid), or specific mannose residues or one or more high mannose type oligosaccharides can be modified with phosphate groups. The latter process leads to the formation of M6P residues, which is catalysed by two enzymes, sequentially. The first enzyme involved is N-acetylglucosaminyl-1-phosphotransferase (GlcNac-1-phosphotransferase), which transfers the GlcNac-1-phosphate from UDP-GlcNac to select C6-hydroxyl groups of mannoses, generating phosphodiester forms. The second enzyme is the N-acetylglucosamine-1-phosphodiester α N-acetylglucosaminidase, also referred to as uncovering enzyme (UCE). This is a type I membrane protein mainly localized to the *trans*-Golgi network (TGN), which cycles constitutively via the plasma membrane. UCE-catalysed hydrolysis of the N-acetylglucosamine-1-phosphodiester on high mannose type oligosaccharides exposes M6P residues. These M6P residues are also found on proteins which haven’t been assigned lysosomal functions or localization, potentially being the cause of lysosomal storage diseases of unknown aetiology [14].

When the hydrolases arrive at the TGN, their M6P tag is identified by M6PR, which are type I transmembrane glycoproteins [15] (Figure 1).

M6PR sorting at the TGN was thought to be predominantly dependant on binding to the heterotetrameric adaptor-protein complex AP-1, which also mediates clathrin recruitment [16]. However, this notion was altered with the discovery of the Golgi localized, γ-ear-containing, ARF-binding family of proteins (GGA) such as clathrin adaptors [17,18]. GGAs are monomeric proteins, which function in parallel with AP-1 to produce M6PR-containing vesicles at the TGN. Thus, the delivery to the endosomal compartments is possible. Alternatively, or additionally, by interacting with AP-1, GGAs are thought to facilitate M6PR entry into clathrin-coated vesicles [19].

The M6PR-ligand complexes exit the TGN in clathrin-coated vesicles, which fuse with endosomal structures. The early endosomes (EEs) receive the lysosomal hydrolases bound to M6PR in vesicles from the TGN [15]. In the acidic environment of the EE, the enzymes dissociate from M6PR and remain in the endosomal lumen. In contrast, the M6PR can follow one of two routes: entering specialized recycling carriers (the endosome to TGN carriers) or go into a tubular sorting endosome (TSE)—an extension of the EEs—where additional recycling carriers exit. The first type, endosome to TGN carriers, are formed directly from endosomal vacuoles and allow M6PR to return to the TGN for other cycles of transport (Figure 1). As for the TSE, it can carry proteins for recycling (to the plasma membrane or to the TGN), as well as low levels of lysosomal membrane proteins (LMP), such as lysosome associated membrane proteins—LAMP1 and LAMP2, designed for the lysosome [15].

Regarding the hydrolases, these are then transported to the lysosomes through the endosomal intermediates [15]. Nevertheless, some hydrolases tagged for endosome/lysosome delivery do not actually arrive at their destination. These enzymes escape M6PR tagging in the TGN and are transported, by default, to the cell surface, where they are secreted into the extracellular fluid. In such cases, various M6PR can deviate back to the plasma membrane and recapture the hydrolases that escaped. Finally, the M6PR deliver them back to the lysosomes by receptor mediated endocytosis via early and late endosomes [20,21]. Numerous studies have shown the existence of M6PR-independent transport of lysosomal proteins, which can include alternative receptors. Sortilin, also named neurotensin receptor 3, plays a role in mediating lysosomal trafficking of prosaposin and sphingomyelinase. Regarding prosaposin, the newly synthesized M6P-containing polypeptide is mostly secreted and reinternalized by the low density lipoprotein receptor-related protein (LRP). In addition, the lysosomal integral membrane protein 2 (LIMP-2) has also been identified as a specific receptor for β-glucocerebrosidase [14]. In the absence of specific targeting characteristics, the lysosomal proteins from the TGN may follow the constitutive secretory pathway to the plasma membrane and subsequently reach lysosomes by endocytosis [15] (Figure 1). The constitutive secretion is the default pathway used primarily to replenish material at the plasma membrane [14].

#### 2.1.2. The Endocytic Pathway

Through several endosomal intermediates, the endocytic pathway delivers macromolecules for degradation to the lysosomes. This pathway begins with the fission of the plasma membrane, giving rise to early endosomes (EE) (Figure 1). The route’s endosomal intermediates undergo maturation, where they are remodelled into later stage endosomes [22]. The maturation process entails several kiss-and-run events and complete fusion events, with constant input and output of membranes and the trade of material through vesicular transport [23].

The EE’s role is to sort the material for recycling and degradation, which can either be received from the plasma membrane and then go back to it or be transported further along the pathway. Thus, EE hold a vacuolar sorting endosome (VSE) part, with a few intraluminal vesicles (ILVs) in which the material destined for lysosomes usually remains [15]. From the EE’s vacuole emerges the TSE (a reticulum of multi branching tubules), where cargo to be recycled and proteins without specific targeting are further sorted [24].

After the maturation events, the now late endosomes/multivesicular bodies can either release their content to the extracellular space or fuse with lysosomes [25,26]. In the first process, late endosomes or multivesicular bodies fuse with the plasma membrane and release ILVs, now termed exosomes, to the cell exterior [26,27,28]. Exosomes can deliver a variety of molecules to other cells, such as miRNA, and influence a myriad of biological functions [29,30]. The second process, which is the fusion of late endosomes with lysosomes, requires a variety of proteins. These include cytosolic factors, like NSF (N-ethyl-maleimide-sensitive factor), soluble NSF-attachment proteins, the small GTPase Rab7, tethers made up of the homotypic fusion and vacuole protein sorting (HOPS) proteins and trans-SNARE (soluble N-ethylmaleimide-sensitive factor-attachment protein receptor) complexes. In addition, calcium released from the lumen of the fusing organelles seems to be a key component in the fusion process [31,32].

Following fusion, if no recovery process occurs, lysosomes and late endosomes will be consumed. To prevent this, lysosomes can be reconstructed from endolysosomes (EL) through a maturation process, which entails content condensation and retrieval pathways. Regarding lysosome content condensation, it has been proved that it requires a proton-pumping ATPase and luminal calcium to generate dense-core lysosomes. Thus, the ATPase in the lysosomal membrane is not only responsible for creating the acidic environment for macromolecule hydrolysis by lysosomal hydrolases, but also for generating dense-core lysosomes [32,33]. As for the membrane retrieval pathways, these are used to remove endosomal membrane proteins and to recycle SNARE. This data has been proven by live-cell microscopy, which has shown vesicular tubular structures that leave endolysosomes after endosome–lysosome fusion [34].

A similar process occurs in the re-formation of lysosomes from autolysosomes formed by autophagosome–lysosome fusion [35]. In this process, regulated by mTOR, protolysosomal tubules extrude and mature into lysosomes [36].

These notions are contrary to the initial thought that lysosomes were the terminal degradative compartment of the endocytic pathway. Thus, most degradation is likely to happen in endolysosomes while they are formed and undergo the maturation process of lysosome reformation [31].

#### 2.1.3. Lysosomal Membrane Protein Pathways

The lysosomal soluble and transmembrane proteins are targeted to lysosomes in a signal-dependent manner. As mentioned previously, the majority of soluble acid hydrolases are modified with M6P residues, which are recognized by M6PR in the Golgi complex and then transported to the endosomal/lysosomal system. In contrast, other soluble enzymes and non-enzymatic proteins are transported to lysosomes in an M6P-independent manner. This process is mediated by alternative receptors, such as the LIMP-2 or sortilin.

Lysosomal membrane proteins do not depend on M6PR for sorting, as they are not modified with M6P groups. Instead, the sorting is performed with the aid of sorting signals present in their cytosolic tails that mediate both lysosomal targeting and rapid endocytosis from the cell surface. These signals have been characterized for members of the LAMP/LIMP class but can also be present in other lysosomal membrane proteins [37].

Lysosomal membrane proteins (LMP) are sorted through multiple clathrin dependent and independent pathways. After being synthesized in the ER, LMP are usually transported as glycosylated proteins to the TGN, where they follow the secretory pathway to the plasma membrane and then are re-internalized through endocytosis. As an alternative, some LMP are transported directly from the TGN to the lysosomes through endosomal compartments [38].

For different cell types, there are different exit points in the TGN used by the individual membrane proteins and the cytosolic adapter proteins. Most LMP contain tyrosine or di-leucine based sorting motifs in their cytosolic domains, which interact with heterotetrameric adaptor protein complexes AP1, AP2, AP3 or AP4. These AP complexes can recruit clathrin and thus initiate the assembly and formation of clathrin-coated vesicles. Specific di-leucine signal subtypes also recruit GGA proteins [39].

In addition, evidence suggests that non-clathrin coated pathways can also be involved in LMP export. Different subunits of multiprotein complexes (ex. the V-type ATPase complex) could be independently targeted. Some multi-spanning transmembrane proteins can require several motifs that act synergistically in order to mediate lysosomal targeting [40].

The lysosomal membrane’s protein components are of great importance to the lysosome’s normal functions [41,42]. An example of the protein-mediated transport is the delivery of glucocerebrosidase (GCase) to the lysosome, which involves LIMP-2. GCase linked with LIMP-2 is inactive, but the acidic milieu of lysosomes leads to its dissociation, promoting GCase activation [43] (Figure 1).

Furthermore, LMP may have a role in the lysosome’s fusion and interactions with themselves and with other cell components, such as the plasma membrane, endosomes and phagosomes. Thus, LMP such as mucolipin 1 (TRPML1 or ML1) (which functions as an ion transporter), possess a crucial role in lysosome biogenesis and functions [44,45].

### 2.2. Lysosomal Functions

The lysosome’s role in the breakdown of extracellular particles from endocytosis and intracellular substances from autophagy is well established. The primary defect of the over 60 LDs is often the occurrence of mutations in genes coding for lysosomal hydrolases (proteases, lipases and glycosidases). In addition, LDs may have different underlying causes such as mutations in a vast number of genes encoding non-lysosomal hydrolases, such as proteins necessary for proper lysosomal function like proteins involved in trafficking from the ER to lysosomes, lysosomal transmembrane proteins or specific enzyme activator proteins. In order to perform their roles, enzymes sometimes require specific activators, such as GM2A activator, essential in GM2 degradation, which will be further explained later in this article.

The enzymatic breakdown results in lysosomal catabolites, which exit the lysosomes through specific exporters in its membrane or through vesicular membrane trafficking [15]. The disruption of lysosomal function created by catabolite accumulation can lead to impairment of downstream events, ultimately resulting in cell death.

Finally, genetic mutations that affect the biogenesis, trafficking, or maturation of lysosome-related organelles (LRO) have also been linked to disease [46].

### 2.3. Pathogenesis

#### 2.3.1. The Lysosome as a Signalling Hub

Cells constitute a tightly regulated network, controlled by inter-organelle signalling and communication. All of the cell’s constituents play defined, but adaptable roles, having core functions as well as secondary roles. Besides their important role in transport, transmembrane proteins also play a critical role in the transport and recycling of metabolites and ions [47].

#### 2.3.2. Lysosomal Nutrient Sensing and mTORC1 Signalling

The lysosome has long been known as the main mediator of cellular catabolism due to its recycling and degradation functions. However, only recently did research on the mechanisms that underlie lysosomal nutrient sensing became a topic of major interest in the biology of the lysosome. A major advance in this field was the discovery of the mammalian target of rapamycin complex 1 (mTORC1), a nutrient-regulated mechanism that shares a dynamic mechanism with the lysosome and is a major regulator of cellular biosynthetic pathways under specific conditions [48].

mTORC1 supports cell anabolism and growth in the presence of nutrients and growth factors, while inhibiting catabolic pathways, such as autophagy through the phosphorylation of Unc-51- like kinase 1 (ULK1) [49]. It is particularly important that, during autophagy, mTORC1 also regulates lysosome re-formation, a process that helps to restore a full complement of functional lysosomes during starvation periods [36].

Activation of mTORC1 requires its dynamic recruitment to the lysosomal surface, which is mediated by the amino acid-dependent activation of heterodimeric RAG GTPases and their interaction with Ragulator [13,50,51]. The RAG-dependent recruitment of mTORC1 to the lysosomal surface can also be induced by cholesterol through the involvement of cholesterol-binding Niemann–Pick type C1 protein (NPC1) [51,52]. Recently, a lysosomal cholesterol sensing protein (G protein–coupled receptor 155), named LYCHOS has been identified. LYCHOS’s lysosomal membrane localization allows it to sense the levels of cholesterol and to relate the concentrations to the mTORC1-dependent anabolic signalling. In situations of high concentration, cholesterol binds to LYCHOS, which will bind and sequester GATOR1 (activating protein for RAG GTPases) which will then recruit mTORC1 to the lysosome, finally promoting cell growth [52].

Along with mTORC1, RAG GTPases modulate the lysosomal recruitment of other nutrient-sensing molecules, including the tuberous sclerosis complex (TSC) [19], and folliculin (FLCN)–folliculin-interacting protein 1 (FNIP) complexes [20], as well as TFEB [21], a master modulator of lysosome biogenesis and autophagy [7], all of which are mTORC1 regulators. Additional specifications of lysosomal nutrient sensing and on the regulation and functions of mTORC1 have been explored in detail in other review papers [48,53,54].

#### 2.3.3. Lysosomal Calcium Signalling

Lysosomal calcium is crucial for several functions. Calcium trafficking is required for the fusion of lysosomes with other cellular structures, including endosomes, autophagosomes and the plasma membrane [23,55,56], thereby regulating endocytic membrane handling, autophagy and membrane damage repair [57].

Furthermore, the lysosomal release of calcium is involved in the formation of contact sites with the ER, important to maintain an equilibrium in lysosomal calcium [24].

Calcium homeostasis is also vital for lysosomal acidification, required for the activity of lysosomal hydrolases [31,58].

In the lysosomal membrane of mammalian cells three main types of calcium channels exist: transient receptor potential cation channels of the mucolipin family (TRPML), two-pore channels (TPC) and the trimeric calcium two transmembrane channel (P2X4) [23,55]. Some of these channels are found exclusively on endolysosomes, although others have additional locations.

Calcium channels respond to a variety of stimuli, lysosomal calcium channels are influenced and respond to changes in pH, nutrients, cellular stress, small molecules such as ATP, phospholipids and sphingosines, suggesting that their activities can be differentially modulated allowing highly selective calcium signalling responses that are adapted to the cell’s requirements [57].

Conceivably, mucolipin 1 (TRPML1 or ML1), is the best characterized lysosomal calcium channel. TRPML1 mediates calcium release from the lysosomal lumen to the cytosol and can be activated by a number of stimuli, including starvation [59,60] and reactive oxygen species [61]. TRPML1 is also activated by a specific phosphoinositide, phosphatidylinositol 3,5-bisphosphate, which links lysosomal calcium signalling to intracellular trafficking processes [59,62,63]. Interestingly, the gene encoding TRPML1 is mutated in mucolipidosis type IV, a rare neurodegenerative lysosomal disorder [64,65].

TRPML1-mediated calcium release regulates: Lysosomal exocytosis and plasma membrane repair [38], autophagosome–lysosome fusion [32], endosome–lysosome fusion, lysosome size [39] and lysosome re-formation from hybrid organelles following fusion [40]. Cumulatively, the activity of TRPML1 has been associated with specific cellular processes in immune cells, including large particle phagocytosis [66], as well as fast and directional migration of dendritic cells through activation of the actin-based motor protein myosin 2 [67]. TRPML1 is also involved in a positive-feedback loop with TFEB, in which TRPML1 regulates TFEB phosphorylation and subcellular localization, while TFEB regulates the expression of the TRPML1 gene [68].

Moreover, TRPML1 mediates the intracellular clearance of accumulating substrates in LD that is promoted by TFEB [68,69,70]. All, these regulatory functions make TRPML1 a desirable target for pharmacological modulation in various diseases [70,71,72].

#### 2.3.4. Lysosomal Adaptation

Environmental indications, or signals, influence the cellular energy metabolism leading to lysosomal adaptation to the changing environment in order to sustain homeostasis. The question that arises from this premise is how the cell manages to modulate the function of an organelle. Interestingly, the combined approach using TFEB ChIP-seq and overexpression data with promoter sequence analysis and co-expression meta-analysis allowed the identification of ‘CLEAR’ (‘coordinated lysosomal expression and regulation’) [11], CLEAR includes a transcriptional gene network with genes involved in different aspects of lysosomal function and autophagy, TFEB belongs to the MiT-TFE family of helix–loop–helix leucine zipper transcription factors together with MITF, TFE3 and TFEC [73]. TFEB together with the CLEAR gene network play a major role in the control of lysosomal function and autophagy [11,12,74,75].

#### 2.3.5. TFEB and the CLEAR Gene Network

TFEB is an autophagy controller regulator of lysosomal function which regulates genes involved in multiple steps of autophagosome biogenesis, autophagosome–lysosome fusion, lysosome positioning, lysosomal proteostasis, lysosomal degradation pathways, and lysosome exocytosis [12,70,74,76,77].

Thus, TFEB behaves as a master regulator of autophagic flux by controlling load delivery and macromolecule degradation, it is this balancing capability that gives it the role of master regulator [78].

Curiously, TFEB overexpression promotes lysosomal biogenesis and therefore an increase in the number of lysosomes. This aspect may have a crucial role in a disease perspective in which lysosomal function is required to be perfect since older lysosomes may be dysfunctional due to accumulation of undegraded macromolecules [11,72,75,79].

#### 2.3.6. Regulation of TFEB by Environmental Signals

Early studies showed that under steady-state conditions, TFEB is located predominantly in the cell’s cytoplasm, and that its localization depends on nutrient availability; upon starvation TFEB translocate to the nucleus [12,68].

During starvation, calcineurin is activated by lysosomal calcium release through the calcium channel TRPML1, leading to TFEB dephosphorylation and subsequent nuclear translocation [68].

Most importantly, the lysosome possesses a function of nutrient availability sensing that has become increasingly better understood [80]. To perform this ordered role, the lysosome has nutrient-sensitive signalling molecules, such as TFEB and mTOR. mTOR binds to other proteins and is used as a core component of two different protein complexes, mTOR complex 1 (mTORC1) and mTOR complex 2 (mTORC2) [81].

When in a starvation state, mTORC1 dissociates from the lysosome. This allows the efflux of calcium from the lysosome through mucolipin 1, leading to the activation of calcineurin (a phosphatase). Calcineurin can undergo dephosphorylation of TFEB, which in turn translocate to the nucleus to upregulate lysosomal gene expression, such as genes encoding lysosomal proteins. In contrast, when in a fully fed state, the mTORC1 scaffolding proteins (Rag GTPases, the Ragulator protein complex, and p62) are recruited to the lysosome surface. The collective action of these proteins consequently leads to mTORC1 translocation from the cytosol to dock to the late endosomes/lysosomes surface platform. Thus, mTORC1 is activated and able to phosphorylate TFEB, which remains in the cytosol and down-regulates lysosomal gene expression. Additionally, mTORC1 also phosphorylates mucolipin1, lowering the calcium efflux necessary for calcineurin activation. Collectively, these actions allow the cell to adjust to changes in environmental metabolic demands [65,81].

## 3. From Order to Disorder

### 3.1. Lysosome Dysfunction in Disease

The rapidly expanding knowledge of lysosomal function has resulted in a better understanding of how lysosomal defects can lead to disease. While initially lysosomal dysfunction was identified in rare inherited conditions such as LD, more recent studies have revealed a crucial role of defective lysosomal function in common disease entities such as neurodegenerative diseases [82], cancer [83] and metabolic disorders [84]. In particular, a decline in lysosomal function with age has been proposed to explain the prevalence of these diseases in elderly individuals [83,84,85,86,87,88].

In order to illustrate how lysosomal disorder translates into lysosomal diseases we have selected a few examples of puzzling diseases (Table 1).

#### Lysosomal Storage Disorders

Dysfunctional lysosomes cause human disease, this fact is clearly illustrated by the existence of LDs, a group of inherited monogenic diseases characterized by a progressive, multisystemic phenotype. The majority of these diseases are autosomal recessive which results in variable penetrance and in a wider symptom presentation and severity of clinical symptoms.

Molecular lesions in genes encoding lysosomal or non-lysosomal proteins, involved in lysosomal functions, lead to an impairment of lysosome-mediated degradation and recycling processes, with progressive lysosomal accumulation of undegraded substrates [3,47,89,90,91,92].

Mutations in a wide range of genes coding for lysosomal proteins and for several non-lysosomal proteins required for lysosomal function leads to LD. Specific enzyme deficiencies, conduct to a specific type of accumulation in the lysosome, these materials can be sphingolipids, glycoproteins, mucopolysaccharides or other macromolecules. In addition, a number of materials, which are not directly related to the enzymatic defect, also accumulate in several LDs. The disruption of lysosomal function due to additional storage can trigger downstream events that affect the ability of the cell to function properly being abnormalities in signalling, defects in calcium homeostasis, oxidative stress, and inflammation [93].

At first, the pathogenesis of LDs appeared straightforward and directly related to the primary storage of accumulating lysosomal substrates. However, the failure to establish simple correlations between phenotype and genotype and intra-familial variability, led to studies based on genomic, cell biology and pathophysiology approaches and identified secondary pathways with crucial roles in the disease pathogenesis. Several studies showed that in most LDs there is a block of autophagy due to impaired fusion between autophagosomes and lysosomes [94,95].

This block is caused by higher cholesterol accumulation in the lysosomal membrane and consequent reduction of the sorting and recycling of SNARE [96]. Therefore, cells exhibit secondary accumulation of autophagy substrates such as aggregation-prone proteins and altered mitochondria, leading to inflammation and neurodegeneration, which are late-stage features of many LDs [97].

As previously stated, we will be classifying and characterizing LDs using the protein deficiencies that cause them as lysosomal hydrolase deficiency, integral membrane proteins deficiency, enzyme modifiers, activator deficiency, lipid and ion transporters deficiency. Firstly, we will briefly describe the functions and some characteristics of the altered protein in each disease. After that, we will clarify how each LD pathological mechanisms can lead to the phenotypes observed.

### 3.2. Lysosomal Hydrolase Deficiency

**Figure 2:**
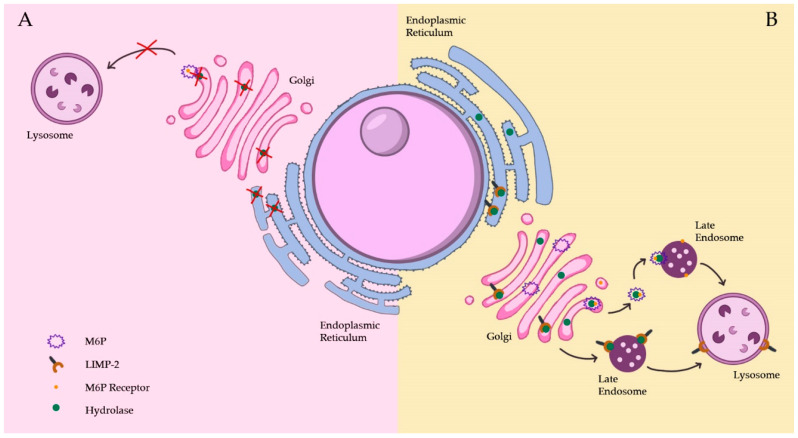
Examples of lysosomal hydrolase deficiency. In (**A**), the mutated hydrolase is not able to pair with the transporter and consequently is not able to reach the lysosome. In (**B**), the mutated hydrolase is carried to the lysosome through the M6P pathway or through the LIMP-2 pathway but is not able to degrade, leading to substrate accumulation in the lysosome.

Over 70 distinct diseases have been included in the group of LDs [3], of which the most common are storage disorders. Several processes may hinder lysosomal functions and lead to subsequent cellular disorder and disease. Lysosomal burden may arise due to various defective processes such as transport, hydrolysis capability or other of the previously mentioned roles of the ordered lysosome (Figure 2). However, in the majority of cases, deficiency in lysosomal hydrolases is the cause of lysosomal storage. LDs present a multisystemic degenerative clinical phenotype, and many are associated with neurodegeneration.

LDs may be caused by mutations in genes codifying lysosomal hydrolases, with subsequent accumulation of a primary type of undegraded substrate. LDs belonging to this category share biochemical and cellular similarities [3,47,89,91,92].

However, it is also possible that a single mutated gene may result in several impaired hydrolases, as examples we can consider the post-translational deficiencies in Multiple sulphatase deficiency (MSD) formylglycine-generating enzyme (FGE) deficiency (due to Sulfatase Modifying Factor 1 (SUMF1) mutations [95]) and in PSAP, (a precursor polyprotein that requires post-translational cleavage and results in four small activator proteins [98,99]). Adding to the complexity, single diseases may be due to mutations in multiple genes, namely Gaucher Disease (GD) and GM2 AB represent examples of this situation in which transport, acidic hydrolase or activator protein deficiencies result in similar phenotypes.

To illustrate a lysosomal hydrolase deficiency, we will focus on GD, in which GCase is affected. We chose this disease due to the recent reports of new variants which break the typical classification of this disease [100].

GCase is a lysosomal enzyme that hydrolyses the sphingolipid glucosylceramide (GlcCer), a cell membrane component, into ceramide and glucose. In GlcCer hydrolysis, Saposin (Sap) C is an essential cofactor that enhances the activity of GCase [101].

The prime cause of GD are mutations in the *GBA1* gene, that encodes GCase. These mutations lead to protein misfolding in the ER, followed by premature degradation by the proteasome [101]. However, LIMP-2 mutations can also affect the GD phenotype, by impairing GCase transport and delivery to the lysosomes [43,102,103]. Infrequently, GD can be also due to a mutation in the *PSAP* gene, which leads to Sap C deficiency, while GCase remains unaffected [104,105].

GCase impairment causes the accumulation of its substrate, GlcCer, in macrophages, due to their phagocytic nature. These cells are particularly affected because they eliminate erythroid cells and leukocytes, which contain large quantities of glycosphingolipids, a source of GlcCer. Consequently, these macrophages develop a foamy appearance, being designated as Gaucher cells, and then infiltrate the bone marrow, the spleen, liver and other organs [106]. Furthermore, the extensive accumulation of GlcCer in the spleen, liver, lung and bone marrow, often leads to chronic inflammation [107]. Gaucher cell infiltration leads to the disease phenotype, which is variable, but with three identified clinical forms. Type 1 (non-neuronopathic) is the most observed form, with no existing neurological damage, while in types 2 and 3 there is neurological impairment present (neuronopathic) [108]. It has been demonstrated that activation of complement C5a controls GlcCer storage and the inflammatory response in Gaucher disease discloses a new potential target for therapy [107].

Clinically, GD can present itself by hepatomegaly, splenomegaly, anaemia, thrombocytopenia, bone lesions or signs of neurological involvement [109]. Regarding neuronopathic GD, Gaucher cells and perivascular or region-specific gliosis can be observed in all types. The gliosis can be present in the hippocampus, parietal cortex and occipital cortex, where neuronal loss is also observed [110].

Despite the identified types of GD, it has been proposed that this disease be viewed as having a continuum of phenotypes, due to the cases where the classification is unclear. For example, in type 1 GD (considered non-neuronopathic), neurological symptoms were observed, due to spinal compression fractures, making the boundaries between types blurry [108].

### 3.3. Integral Membrane Protein Deficiency 

As an example of an integral membrane protein deficiency, we will be examining Danon disease (DD). This disorder was particularly chosen because of its inheritance pattern, which is X-linked and dominant. This pattern is distinct from others observed in lysosomal diseases, which are mostly autosomal and recessive [111].

Lysosome associated membrane proteins 1 and 2 (LAMP-1 and LAMP-2) are major protein components of the lysosomal membrane, contributing to about 50% of all lysosome membrane proteins [112]. Structurally, LAMPs are characterized by a single transmembrane domain, a large, heavily glycosylated luminal domain, and a short C-terminal cytosolic tail [113]. Because of their abundance and membrane location, LAMPs were regarded as a protective barrier from the lysosomal lumen. However, recent advances proved that the LAMPs’ combined action is necessary for autophagy regulation [114].

LAMP-2 takes part in chaperone-mediated autophagy [115], cholesterol-transport [116] and is implicated in MHC class II presentation of cytoplasmic antigens [117]. 

There are three LAMP-2 isoforms, LAMP-2A, -2B, and -2C, generated by alternative splicing of exon 9 in the LAMP-2 gene. These isoforms differ in the transmembrane and cytoplasmic domains but have identical luminal domains [118].

Mutations in the LAMP-2 gene lead to loss of LAMP-2 expression, causing DD, whose mechanism is not fully comprehended [119]. Most LAMP-2 gene mutations result in the deficiency of all three isoforms, causing DD. However, it is suggested that DD is largely caused by defects in the LAMP-2B isoform, because of the existence of patients with the full set of DD symptoms, presenting only a LAMP-2B specific mutation [120]. In addition, LAMP-2B is normally expressed in a tissue-specific manner, being more abundant in the heart and skeletal muscle [121]. The triad of symptoms that characterize the disease: cardiomyopathy, skeletal myopathy and cognitive deficit, is consistent with the –2B isoform preferred location [89].

Because of LAMP-2’s function in autophagy, recent studies have also shown that DD could be caused by a block in autophagy, which would lead to impaired autophagosome-lysosome fusion and/or inefficient lysosome biogenesis and maturation. This is consistent with patients’ increases in cytoplasmic vacuolation, hypertrophic cardiomyocytes, and the degeneration of the myocardium, as evidenced by the accumulation of lipofuscin, an indicator of damage to organelles such as lysosomes [120].

### 3.4. Lipid and Ion Transporters Deficiency

As a representative of lipid and ion transporters deficiencies, type IV mucolipidosis was chosen because it causes dysfunction in calcium mechanisms crucial to the cell. Along the lysosome’s endocytic pathway, there are several calcium-regulated steps, such as the fusion processes [122]. Here, calcium is released from late endosomal/lysosomal lumen compartments via calcium channels, possibly mucolipin 1, a membrane protein encoded by the MCOLN1 gene [63].

Mucolipin 1 protein, or TRPML1, is part of the transient receptor potential (TRP) family of proteins, with a similar structure of six transmembrane domains. Within the TRP family, Mucolipin 1 belongs to a subfamily called the mammalian mucolipin TRP (TRPML), made up of endolysosomal cation channels [90].

Mucolipin 1 is a non-selective calcium permeable cation channel which is ubiquitously expressed, but with highest expression levels in the brain, kidney, liver, spleen, and heart [123].

Loss-of-function mutations in the MCOLN1 gene causes Mucolipin 1 deficiency, which gives rise to type IV mucolipidosis (ML IV). This disease is characterized by an impaired endocytic pathway and an altered mTORC1/TFEB signalling axis [65].

Regarding the impairments in the endocytic pathway, the processes altered are the fusion of late endosomes and lysosomes. The absence of Mucolipin 1 causes defects in the calcium dependent fusion events, leading to enlarged late endosomes/lysosomes. The loss of Mucolipin 1 also leads to improper fission events, which are necessary for the biogenesis of lysosomes and retrograde transport vesicles [123].

ML IV also comprises pathogenic mechanisms underlying the master cell regulator mTOR and the coordinated lysosomal expression and regulation network activator, TFEB [10]. The mucolipin 1 deficiency observed in ML IV creates a calcium deficit. Thus, calcineurin cannot be activated by calcium, which in turn compromises TFEB dephosphorylation and its translocation to the nucleus. Consequently, the cell is unable to respond to a starvation state via TFEB-mediated activation of lysosomal genes [10].

Collectively, these impaired mechanisms result in the disease’s characteristic symptoms such as profound psychomotor disability resulting in hypotonia (and sometimes spasticity), and severe unrelenting cognitive impairment [65].

### 3.5. Enzyme Modifiers and Activator Deficiency

For the last category of LD, GM2 gangliosidosis, AB variant (MIM 272750) was chosen as it exemplifies a rare cofactor deficiency and a rare type of LD [124].

Glycosphingolipids are cell membrane components and the major glycolipids of animals [125]. These glycosphingolipids are degraded to ceramide by lysosomal enzymes through several steps, where small glycoprotein cofactors are also necessary [126].

The degradation of GM2 ganglioside (a sialic-acid-containing glycosphingolipid) requires the catalytically active β-hexosaminidase A (β-HEXA) [127]. β-HEXA is a lysosomal enzyme with two subunits, α and β, which requires the GM2 activator (GM2A), a glycolipid binding protein, to perform its function. The GM2A forms a complex with GM2 ganglioside, extracting it from the membrane, making it accessible to β-HEXA and thus, enabling its degradation [128].

A deficiency in any of the three protein components required for GM2 degradation (β-HEXA subunits α and β or GM2A) leads to the intralysosomal accumulation of GM2 and related glycolipids, primarily in neuronal cells, resulting in a form of GM2 gangliosidosis [129]. Defects in the GM2 activator protein (with normal β-HEXA) cause what is known as GM2 gangliosidosis AB variant [130] (p. 2). This very rare form of the disease is associated with autosomal recessive mutations in the GM2A gene and characterised by cytoplasmic inclusions of storage material in neuronal cells. The clinical phenotype is similar to the one of Tay Sachs, patients are not dysmorphic, they present neurologic symptoms at a very young age, with delayed motor milestones, increasing weakness and ophthalmologic examination usually reveals the presence of cherry-red spots on the macula [129].

LDs may also be caused by mutations in genes that encode non-lysosomal proteins. Such proteins may interfere with various functions, including post-translational modification of lysosomal enzymes. For example, mucolipidosis type II is caused by mutations in the gene that encodes N-acetylglucosamine-1-phosphotransferase, which participates in the mannose 6-phosphate-mediated trafficking of lysosomal enzymes [121,131]. Multiple Sulfatase Deficiency (MIM 272200) is a deficiency due to a post-translation modifier enzyme encoded by the SUMF1 gene (Sulfatase Modifying Factor 1). The formylglycine-generating enzyme (FGE) is an ER resident enzyme and acts upon all sulfatases converting a highly conserved cysteine residue, in their catalytic domain, to C-α-formylglycine [95,131]. This is an example of a known post-translational modification required for the activation of a group of enzymes, the sulfatases. Alterations in the SUMF1 gene lead to complex phenotypes, affect the activity of all sulfatases and result in the accumulation of several sulphated substrates [131,132,133].

**Table 1:** Examples of Lysosomal Storage Diseases with different primary defect mechanisms.

Lysosomal Disorder	Genetic Mutations	Primary Defects	Cellular Consequences
Gaucher Disease (Autosomal recessive)	GBA1	GCase impaired hydrolytic activity or premature degradation [134]	GlcCer accumulation in macrophages, which become Gaucher cells [135] and infiltrate several organs; GlcCer burden is associated with tissue inflammation processes [107].
	LIMP-2	Impaired GCase transport [43,103]	
	PSAP	Saposin C deficiency and impaired GCase function [9,104,105]	
Danon Disease (Dominant X-linked)	LAMP-2	LAMP-2A dysfunction [119]	Accumulation of immature autophagic vacuoles [136]. Glycogen accumulation in autophagic vacuoles [137]. Block in autophagy leads to impaired autophagosome–lysosome fusion and/or inefficient lysosome biogenesis and maturation [138].
Mucolipidosis IV (Autosomal recessive)	MCOLN1	Mucolipin-1 absence [65]	*Altered endocytic pathway* ➢ Decreased fusion of late endosomes and lysosomes, altered calcium signalling, presence of larger acidic organelles [139,140]. ➢ Normal lysosomal hydrolases [140].
			*Altered mTORC1/TFEB signalling axis* ➢ Inability to respond to a starvation state via TFEB-mediated activation of lysosomal genes [122].
GM2 Gangliosidosis AB Variant (Autosomal recessive)	GM2A	GM2 activator deficiency, lack of formation of beta-hexosaminidase A/ GM2 complex [9,130]	Deficient GM2 removal from membrane, deficient degradation leads to the intralysosomal accumulation of GM2 and related glycolipids.

## 4. Concluding Remarks

The discovery and comprehension of the diverse functions of lysosomes revealed that this organelle is not only a waste disposal system, but also a highly complex key cellular signalling component. Due to these discoveries, the rare lysosomal diseases have increasingly gained significance through the years. Through the understanding of lysosome biology and its functions, it is possible to have a better understanding of the lysosomal disease-causing mechanisms. However, despite the light shed through the years in the field of lysosomal diseases, it is clear that some mechanisms are still not fully understood. For example, in GD, future investigation may be directed towards understanding the different organ involvement. The reason why some GD patients present neurological consequences while others do not is still unclear. In addition, in DD, it is crucial to understand how LAMP-2 deficiency leads to the disease’s phenotypes, since the pathological mechanisms are only hypothesis, which are not fully understood. In our opinion, there is a strong need for comprehension of the exact abnormalities in the pathological pathways in lysosomal disease affected cells.

## Data Availability

Not applicable.

## Abbreviations

AP-#	Adaptor Protein #
ARF	ADP ribosylation factor
β-HEXA	β-hexosaminidase A
Ca^2+^	Calcium
CLEAR	Coordinated lysosomal expression and regulation element
DD	Danon Disease
EE	Early endosomes
EL	Endolysosomes
ER	Endoplasmic reticulum
FGE	Formylglycine-generating enzyme
FLCN	Folliculin
FNIP	Folliculin-interacting proteins
GATOR1	GTPase-activating protein (GAP) for RAG A
GCase	Glucocerebrosidase
GD	Gaucher Disease
GGA	Golgi-localized, γ ear–containing, ARF–binding protein
GlcCer	Glucosylceramide
GM2	Ganglioside monosialic 2
GM2A	GM2 ganglioside activator protein
GTPase	GTP (guanosine triphosphate)-binding proteins
HOPS	Homotypic fusion and vacuole protein sorting
ILVs	Intraluminal vesicles
LAMP#	Lysosomal-associated membrane protein #
LD	Lysosomal Disease
LIMP-#	Lysosomal integral membrane protein- #
LMP	Lysosomal membrane proteins
LRO	Lysosome-related organelles
LRP	Lipoprotein receptor-related protein
LYCHOS	Lysosomal cholesterol sensing protein (G protein–coupled receptor 155)
M6P	Mannose 6-phosphate
M6PR	Mannose-6-phosphate receptors
MCOLN1	Mucolipin TRP Cation Channel 1; protein coding gene
MITF	Microphthalmia-associated transcription factor
MiT-TEF	Family of transcriptions factors
ML1	Mucolipin-1
ML IV	Mucolipidosis type IV
MSD	Multiple sulphatase deficiency
mTOR	Mammalian target of rapamycin
mTORC #	Mammalian target of rapamycin complex #
NPC1	Niemann–Pick type C1 protein
NSF	N-ethyl-maleimide-sensitive factor
PSAP	Gene that encodes prosaposin
RER	Rough Endoplasmic Reticulum
Sap C	Saposin C
SNARE	Soluble N-ethylmaleimide-sensitive factor-attachment protein receptor
SUMF1	Sulphatase Modifying Factor 1
TFE3	Transcription factor E3
TFEB	Transcription factor EB
TFEC	Transcription Factor EC
TGN	*Trans*-Golgi network
TPC	Two-pore channel
TRP	Transient receptor potential
TRPML	Transient receptor potential of the mucolipin family
TRPML1	Transient receptor potential of mucolipin 1
TSC	Tuberous sclerosis complex
TSE	Tubular sorting endosome
UCE	Uncovering Enzyme
UDP-GlcNac	Uridine diphosphate N-acetylglucosamine
ULK1	Unc-51- like kinase 1
VSE	Vacuolar sorting endosome
